# Interferon-Gamma Release Assay: An Effective Tool to Detect Early *Toxoplasma gondii* Infection in Mice

**DOI:** 10.1371/journal.pone.0137808

**Published:** 2015-09-17

**Authors:** Qing Yin, Saeed El-Ashram, Hongbin Liu, Ximeng Sun, Xinxin Zhao, Xianyong Liu, Xun Suo

**Affiliations:** 1 State Key Laboratory for Agrobiotechnology, China Agricultural University, Beijing, 100193, China; 2 National Animal Protozoa Laboratory & College of Veterinary Medicine, China Agricultural University, Beijing, 100193, China; 3 Department of Pharmacology, Hebei North University, Zhangjiakou, 075000, China; 4 Department of Parasitology, Capital Medical University, Beijing, 100069, China; 5 Key Laboratory of Animal Epidemiology and Zoonosis of Ministry of Agriculture, Beijing, 100193, China; 6 Faculty of Science, Kafr El-Sheikh University, Kafr El-Sheikh, Egypt; Food and Drug Administration, UNITED STATES

## Abstract

Early diagnosis of *Toxoplasma gondii* infection before the formation of tissue cysts is vital for treatment, as drugs available for toxoplasmosis cannot kill bradyzoites contained in the cysts. However, current methods, such as antibody-based ELISA, are ineffective for detection of early infection. Here, we developed an interferon-gamma release assay (IGRA), measuring the IFN-γ released by T lymphocytes stimulated by *Toxoplasma* antigen peptides *in vitro*, for the detection of *T*. *gondii* infection in mice. Splenocytes isolated from infected mice were stimulated by peptides derived from dense granule proteins GRA4 and GRA6 and rhoptry protein ROP7, and released IFN-γ was measured by ELISA. Results showed that both acute and chronic infection could be detected by IGRA. More importantly, IGRA detected infection as early as the third day post infection; while serum IgM and IgG were detected 9 days and 13 days post infection, respectively. Our findings demonstrated that an IGRA-positive and ELISA-negative sample revealed an early infection, indicating the combination of IGRA and ELISA can be employed for the early diagnosis of *T*. *gondii* infection in human beings, cats and livestock.

## Introduction

*Toxoplasma gondii* is a protozoon that can infect all warm-blooded animals and its infection can cause severe disease in humans and animals at the very beginning of infection [1–3]. Initial infection and acute disease are characterized by the presence of fast-replicating tachyzoites. The host may die of acute toxoplasmosis or recover with the acquisition of immunity [4, 5]. *T*. *gondii* infection elicits a strong Th1 response including with IFN-γ release [6, 7]. Around 10–14 days post infection, tachyzoites differentiate into bradyzoites that replicate more slowly and preferentially form cysts in brain and muscle tissues [8].

Antiparasitic drugs, such as sulfonamides and pyrimethamine, limit proliferation of tachyzoites during active infection [9]. However, parasites cannot be eliminated by these chemotherapeutic agents once they convert into bradyzoites in tissue cysts [8]. Therefore, only early detection of the infection secures an effective treatment [10].

Antibody-based serological tests are currently the most common diagnostic methods for detection of *T*. *gondii* infection [11, 12]. However, *T*. *gondii*-specific IgM and IgG are not detectable until around two weeks post infection [13, 14], which means a seropositive test usually reveals tissue cyst formation.

Interferon-gamma release assay (IGRA), a T-cell-based test, was first introduced as an *in vitro* test detecting *Mycobacterium tuberculosis* infection, and later used for early detection of subclinical pulmonary tuberculosis [15–19]. In the present study, we showed that IGRA can detect both acute and chronic *T*. *gondii* infection and more importantly, it can detect infection as early as the third day post infection.

## Materials and Methods

### 1. Ethics statement

Animal experiments were conducted in accordance with the guidelines of Beijing the Municipality on the Review of Welfare and Ethics of Laboratory Animals approved by the Beijing Municipality Administration Office of Laboratory Animals (BAOLA) and under the protocol (CAU-AEC-2010-0603) approved by the China Agricultural University Animal Ethics Committee. All experimental procedures were also approved by the Institutional Animal Care and Committee of China Agricultural University (The certificate of Beijing Laboratory Animal employee, ID: 15883). The mice were humanely euthanized by cervical dislocation after anesthetization. The mice were anesthetized by subcutaneous injection of Atropine (0.02 mg/kg) before euthanasia. All efforts were made to minimize animal suffering.

### 2. Animals, parasites and antigen peptides

Six- to eight- week-old specific pathogen free (SPF) grade female BALB/c mice were purchased from the Institute of Laboratory Animal Sciences, Chinese Academy of Medical Sciences (Beijing, China). A type II strain of *T*. *gondii* parasites, Prugniaud (Pru), was passaged every 4 to 5 weeks in mice by oral infection with 5 cysts.

Three immunodominant peptides, dense granule proteins GRA4, GRA6 and rhoptry protein ROP7 were selected for the stimulation of *T*. *gondii*-specific T cells in BALB/c mice according to the previous publications [20, 21]. The amino acid sequences of these three peptides are SPMNGGYYM [20], HPGSVNEFDF [21], and IPAAAGRFF[20], respectively. These peptides were synthesized by GL Biochemistry Ltd. (Shanghai, China).

### 3. Experimental design

**Fig 1:**
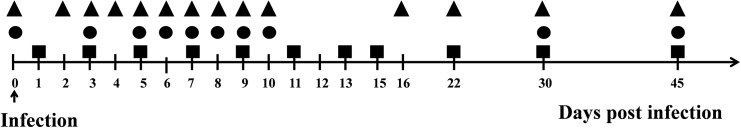
Schematic illustration of the experimental design. Each mouse in the infected group was inoculated intra-gastrically with 4 cysts of Pru strain at day 0. At the indicated days, blood samples (black square) were collected from tail vein and sera were separated for ELISA, while splenocytes were isolated from spleens for ICS (black circle) and IGRA (black triangle).

Each mouse of the infected group (n = 45) was inoculated intra-gastrically with 4 cysts of Pru strain, while the mice of the uninfected group were inoculated intra-gastrically with sterile PBS. Blood and spleens were collected from 3 randomly selected mice at different days post infection (Fig 1). The group became smaller over the course of the experiment. Three independent experiments were performed and results of one representative experiment are presented here.

### 4. Preparation of mice splenocytes

Spleens from *T*. *gondii* infected or uninfected mice were harvested and splenocytes were released by grinding the spleen through a 70-μm-pore-size nylon screen. Splenocytes were pelleted then subject to erythrocyte lysis (Quantobio, Beijing, China) for 3 min at room temperature. Splenocytes were then washed twice in sterile PBS and live cells were identified via trypan blue exclusion and enumerated by using a hemacytometer.

Splenocytes were resuspended in RPMI 1640 medium (HyClone, USA) supplemented with 100U/ml penicillin and 100μg streptomycin (M&C Gene Technology, Beijing, China), 10% heat-inactivated fetal bovine serum (HyClone, USA) and 0.002% β-mercaptoethanol (Sigma, MO, USA).

### 5. Interferon-gamma release assay

Splenocytes of each mouse were divided into 3 portions, and stimulated with either concanavalin A (20μg/ml; positive control), with enriched RPMI 1640 medium (negative control) or with a mixture of three *T*. *gondii*-specific peptides (4 μg/ml/peptide). After incubation for 24 h, at 37°C and 5% CO_2_, supernatant was collected to assess the IFN-γ concentration in duplicate, employing the mouse IFN-γ ELISA kit according to the manufacturer’s protocol (Biolegend, CA, USA).

### 6. Intracellular cytokine staining (ICS) assay

Mouse splenocytes were cultured in 12-well round bottom plates (4×10^6^ cells/well) and stimulated with or without mixed *T*. *gondii* peptides (4 μg/ml/peptide) for 24 h. Cells were treated with BD GolgiStop™ protein transport inhibitor (BD Biosciences, San Diego, USA) for 6 h post stimulation. Then, cells were recovered and blocked with anti-mouse CD16/32 (Biolegend, CA, USA). After washing twice with cell staining buffer (BD Biosciences), cells were prepared in 50μl staining buffer and stained with FITC anti-mouse CD3ε (Biolegend, CA, USA) and with APC anti-mouse CD8α (Biolegend, CA, USA) at 4°C for 30 min. Following two additional washes, cells were thoroughly suspended in 250 μl fixation/permeabilization solution (BD Biosciences) for 20 min at 4°C. Then cells were washed, re-suspended in 50μl of BD Perm/Wash solution and stained with PerCP/Cy5.5 anti-mouse IFN-γ (Biolegend, CA, USA) at 4°C for 30 min. Finally, IFN-γ expression in CD8^+^CD3^+^ T cells was measured by BD Accuri™ C6 flow cytometer.

### 7. ELISA

The serum IgG and IgM levels were measured by indirect ELISA. Tachyzoite antigens were prepared as previously described [22]. Tachyzoite antigens (4μg/ml) were prepared in PBS, and 100 μl solution was added to each well of a 96-well ELISA microtiter plate (Nunc-immuno MaxiSorp plate, Nunc, Roskilde, Denmark). The plates were incubated at 4°C overnight, and then the wells were washed three times with PBS containing 0.05% Tween 20 (AMRESCO Inc., USA). Next, the plates were blocked with PBS containing 5% skim milk (BD biosciences, USA) at 37°C for 2 h and then washed three times. Serum samples were diluted 1:8000 for IgG or 1:5000 for IgM in PBS containing 2% skim milk, and 100μl of this solution was dispensed to duplicate wells. The plates were washed three times after 1h incubation at 37°C. Then 100 μl 1:2000 diluted HRP-labeled anti-mouse IgG (H + L) Ab (Proteintech Group, Inc., USA) or anti-mouse IgM Ab (Biosynthesis Biotech., China) was added to each well and the plates were incubated at 37°C for 1h followed by four time washes. Substrate solution containing TMB A solution and B solution (MACGENE Biotech., China) was added to the wells for 10 min at room temperature. The reaction was stopped by adding 50 μl of 2M H_2_SO_4_. Finally, the plate was read at 450/630 nm by a microplate reader (Bio-Rad Laboratories, Calif., USA).

### 8. Statistics

The antibody levels were expressed as the mean level of 3 mice per group ± S. D. Analysis of all FACS data was performed using the Accuri CFlow software (Accuri Cytometers).

## Results

### 1. IGRA detection of *T*. *gondii* infection

**Fig 2:**
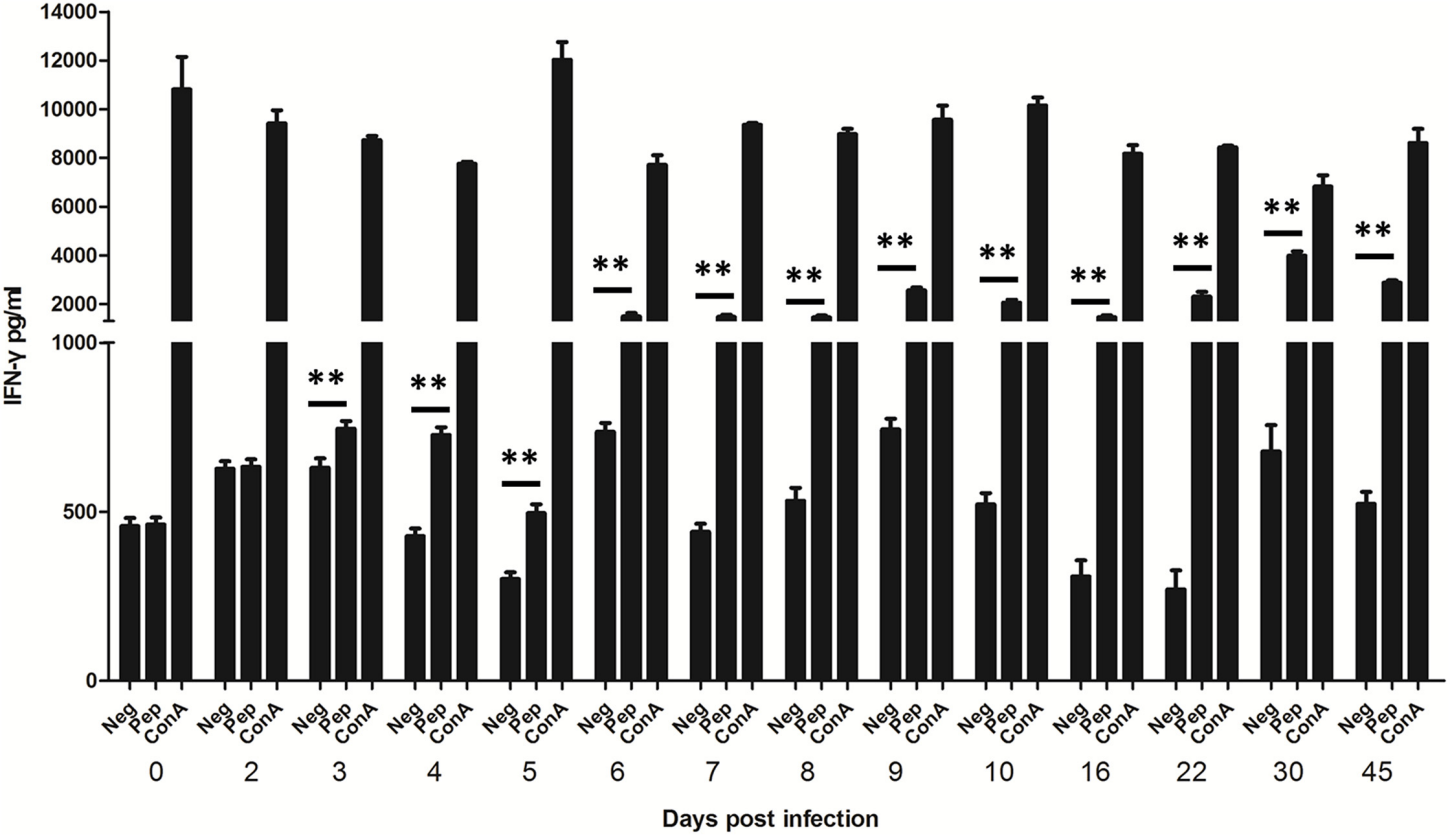
Dynamics of IFN-γ levels in cyst-infected mice detected by IGRA. Mouse splenocytes were collected at 0, 2, 3, 4, 5, 6, 7, 8, 9, 10, 16, 22, 30, 45 dpi, and stimulated with synthesized *T*. *gondii* peptides *in vitro*. Concentration of IFN-γ in the supernatant was detected by ELISA. Neg, negative control; Pep, splenocytes stimulated by peptides; ConA, positive control. Data represent mean values for 3 mice in each day. **p<0.01(different dpi compared with 0 dpi).

In order to determine if IGRA could be used for the early detection, we applied it on mice infected with *T*. *gondii*. Mouse splenocytes were collected at different days post infection and stimulated with synthesized *T*. *gondii* peptides *in vitro*. Concentration of IFN-γ in the supernatant was detected by ELISA. By comparing IFN-γ levels of the peptide-stimulated well and the negative well, *Toxoplasma* infection was detected as early as 3 days post infection (dpi) (*p*<0.01) and as late as 45 dpi (Fig 2).

### 2. ICS detection of *T*. *gondii* infection

**Fig 3:**
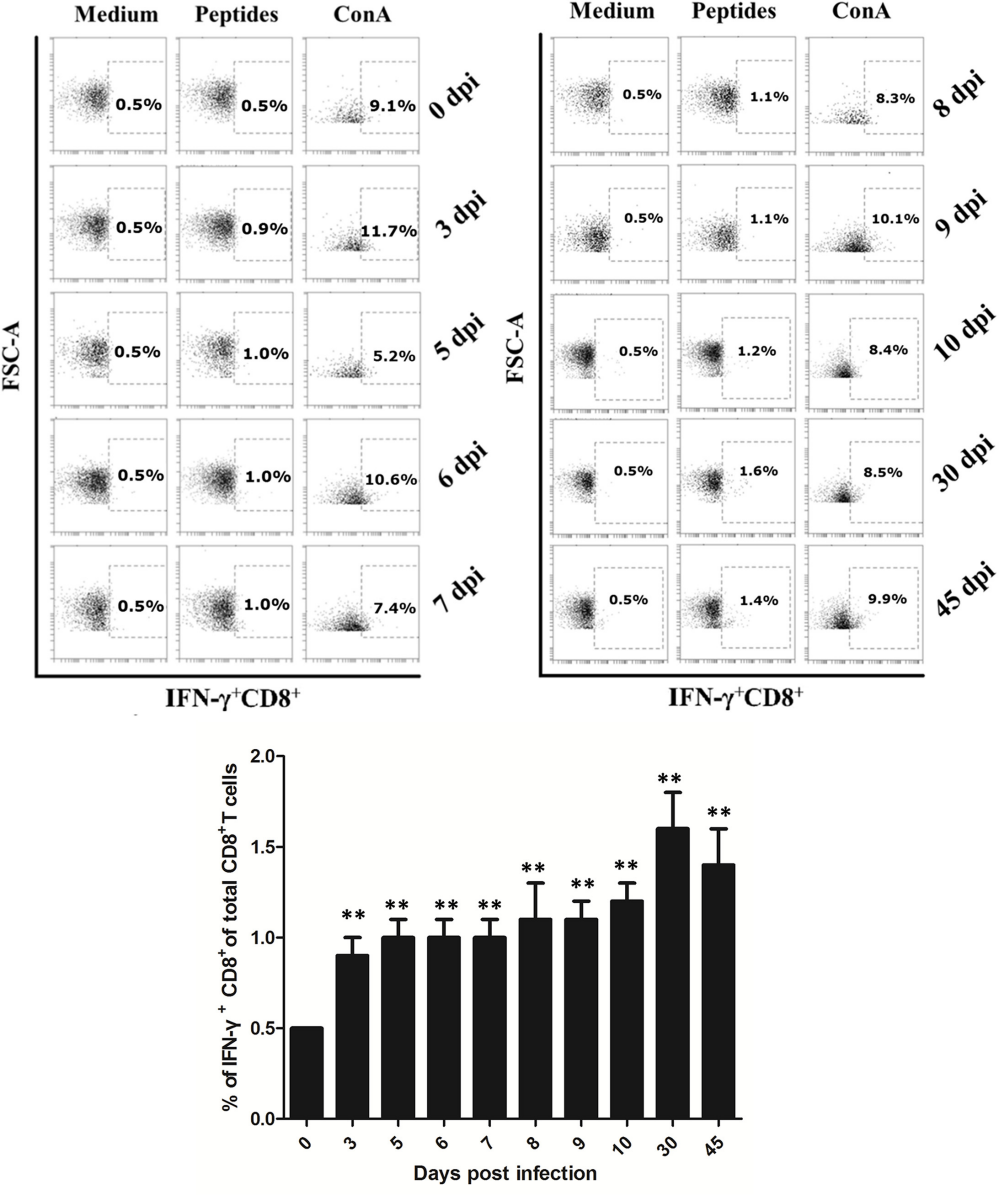
IFN-γ expression in CD8^+^ T cells stimulated with synthesized *T*. *gondii* peptides *in vitro*. (A) Representative flow cytometry plots obtained at day 0, 3, 5, 6, 7, 8, 9, 10, 30, 45 post oral infection with *T*. *gondii* cysts for IFN-γ in CD8^+^ T cells stimulated *in vitro* with three synthesized peptides. Numbers indicate average percent IFN-γ^+^ CD8^+^ T cells in total CD8^+^ T cells. (B) Percent IFN-γ^+^ CD8^+^ T cells within the total CD8^+^ T cells in mouse splenocytes. Data represent mean values for 3 mice in each day. **p<0.01(different dpi compared with 0 dpi).

To verify the reliability of IGRA for the detection of *T*. *gondii* in mice, we compared it with ICS, a standard method for detecting T-cell responses. Mouse splenocytes were collected at different days post infection and stimulated with synthesized *T*. *gondii* peptides *in vitro*. The percentage of IFN-γ releasing CD8^+^ T cells was detected by flow cytometry. Peptide-specific IFN-γ^+^ CD8^+^ T cells was detected at 3 dpi (*p*<0.01), and this percentage continued to increase slowly until the 30 dpi, with a slight decrease from this day to 45 dpi (Fig 3A and 3B).

### 3. ELISA detection of *T*. *gondii* infection

**Fig 4:**
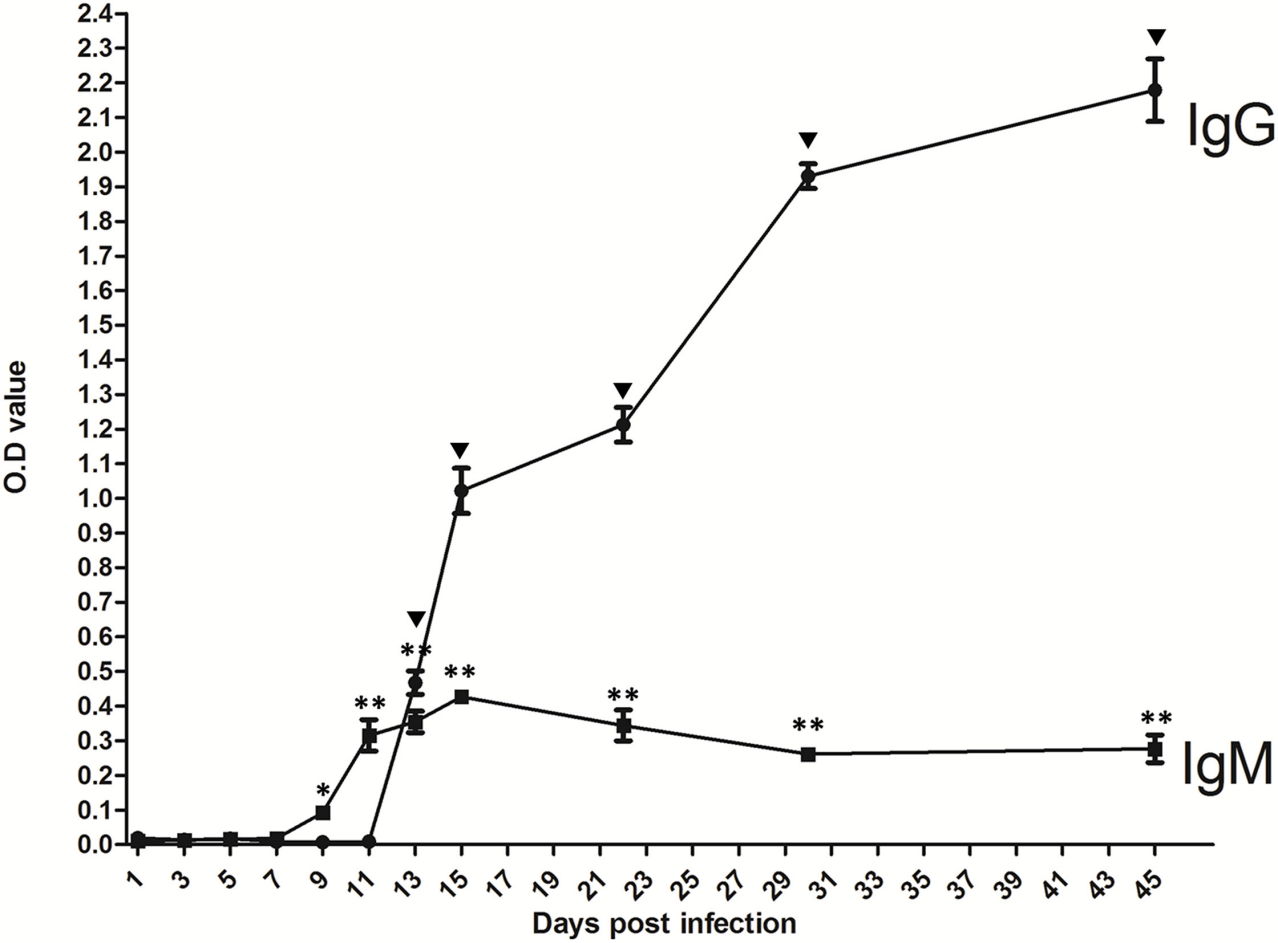
Dynamics of humoral responses in mice after orally infected with *T*. *gondii* cysts. Detection of *T*. *gondii*-specific IgG (A) and IgM (B) in the serum of BALB/c mice (n = 3) infected with four cysts at 1, 3, 5, 7, 9, 11, 13, 15, 22, 30, 45 dpi with ELISA. Results are expressed as mean of the OD 450/630 ± S.D. Inverted black triangle *p*<0.01 (different dpi compared with 1 dpi for IgG), * *p*<0.05, ** *p*<0.01(different dpi compared with 1 dpi for IgM).

To determine the earliest detection time of humoral response, ELISA was applied in infected mice. The IgM was detected at 9 dpi (*p*<0.05). Its level peaked at 15 dpi and decreased slightly from 29 dpi, with the stable level from 30 dpi to 45 dpi (Fig 4A). The IgG was detected at 13 dpi (*p*<0.01), with a continuous increase until 45 dpi (Fig 4B).

## Discussion

In this study, IGRA was developed to detect *T*. *gondii* infection in BALB/c mice after oral infection with cysts. Results showed that IGRA detected both acute and chronic infections during the experimental period, and more importantly, this simple method detected very early infection, as early as the third day post infection.

After exposure to infection, host naïve T cells are activated in a very short time and the IFN-γ released by activated CD8^+^ T cells could be detected at 3 dpi [23–25]. Our finding that IGRA detected *T*. *gondii* infection at the third day post infection is consistent with these data. In contrast, serum IgM and IgG were only detected at 9 days and 13 days after infection by ELISA, respectively. Early diagnosis leads to an effective treatment with higher chances of recovery from toxoplasmosis [8, 9, 26, 27]. Our finding demonstrated that an IGRA-positive and ELISA-negative sample revealed an early infection, indicating a new strategy for the early diagnosis of *T*. *gondii* infection by combining IGRA and ELISA.

Detecting cell-mediated immunity (CMI), like ICS, post infection or vaccination is labor-intensive and requires skilled technicians and expensive instruments [28]. Nevertheless, IGRA, based on ELISA detection of IFN-γ released by *T*. *gondii*-specific T cells, was an easy-operation and low-cost method to measure CMI. Besides, reliability was proved by comparing it with ICS in this study. Our result indicates IGRA is an alternative assay for the evaluation of CMI against *T*. *gondii*. Our results further corroborate the idea of utilizing whole blood-based IFN-γ release assay to diagnose congenital toxoplasmosis [29, 30].

To decrease the risk of bias of using a single stain of mice, and to generalize ELISA-based IGRA to warm-blooded animals, including humans, cats with more diverse genetic backgrounds have been utilized to validate the results. Interestingly, mouse results are consistent with data obtained by using a whole blood IGRA for the early *T*. *gondii*-infected cats [31]. In summary, we developed IGRA, a T-cell-based test, as an early detection method for the diagnosis of *T*. *gondii* infection. Our finding suggests combination of IGRA and ELISA can be employed for the early diagnosis of *T*. *gondii* infection in warm-blooded animals, including humans. Furthermore, ELISA-based IGRA holds the potential to become a useful commercial diagnostic tool for early and chronic detection of *T*. *gondii* infection.
